# Aversion for Avulsion: A Case of Delayed Diagnosis of Traumatic Tricuspid Rupture

**DOI:** 10.1155/2019/1562124

**Published:** 2019-11-05

**Authors:** Wendy K. Bernstein, Nicholas Goehner

**Affiliations:** ^1^Department of Anesthesiology and Perioperative Medicine, University of Rochester Medical Center, Rochester, NY, USA; ^2^Department of Anesthesiology, Kaiser Permanente Wailuku, Maui Memorial Medical Center, Maui, HI, USA

## Abstract

Tricuspid rupture secondary to blunt force trauma is a rare diagnosis. However, the incidence of this injury is rising due to the improved initial treatment of complex trauma patients as well as enhanced early detection methods through the use of cardiac ultrasound. We report the case of an otherwise healthy 42 year old male who sustained significant blunt force trauma after a single motor vehicle accident. The diagnosis of traumatic papillary rupture and disruption of the valve apparatus was made on the day of admission after perioperative hemodynamic compromise and the use of intraoperative transesophageal echocardiography. However, treatment was delayed due to concerns of systemic anticoagulation leading to his eventual demise.

## 1. Introduction

Myocardial valve injuries after blunt force trauma are rare [1, 2]. Thus, the diagnosis of new onset tricuspid regurgitation can be delayed for patients that suffer from a motor vehicle accident (MVA) [3–5]. In addition, many adults can tolerate tricuspid regurgitation, until its severity results in congestive hepatopathy, conduction abnormalities, and right heart failure.

The early diagnosis of myocardial valvular injuries after blunt force trauma may have a profound impact on medical management, and potentially lead to definitive surgical treatment which could prevent cardiovascular collapse [6]. We report the case of a 42 year old male who sustained a high speed single MVA resulting in significant blunt chest trauma. The delayed diagnosis of tricuspid valve injury from traumatic papillary rupture and disruption of the valve apparatus was made after perioperative hemodynamic compromise and was facilitated by the use of intraoperative transesophageal echocardiography (TEE).

## 2. Case Description

The patient was a 42 year-old male with past medical history of obesity, gastroesophageal reflux and alcohol abuse who presented to the R Adams Cowley Shock Trauma Center after a single motor vehicle accident. Initially, he arrived conscious, and alert with glascow coma scale (GCS) 15 despite severe blunt chest trauma from direct impact with the steering column. On initial physical exam, a grade II/VI systolic murmur was heard at the lower left sternal border. His lungs were clear to auscultation bilaterally. A chest radiograph demonstrated a widened mediastinum, multiple rib fractures (T3–T8), sternal fracture and flail chest. Electrocardiogram was normal. Laboratory tests were unremarkable. Lactate level was 3.3 and Troponin levels were 24.4 ng/ml. The CKMB was drawn but results were not available due to hemolysis. An initial focused assessment with sonography in trauma (FAST) and pelvis radiographs were normal.

During assessment, the patient became hypotensive, tachycardic and unresponsive. He was immediately intubated and bilateral chest tubes were placed. A bedside left thoracotomy was performed for suspected pericardial tamponade. Despite fluid resuscitation and intravenous dobutamine, pulseless electrical activity (PEA) arrest occurred. No significant hemopericardium was encountered upon entering the chest. However, hemodynamics improved with a blood pressure 130/73 mmHg, and heart rate 82 beats per minute upon entering the pericardium. The heart was noted to be enlarged with decreased function and right sided contusion.

**Figure 1:**
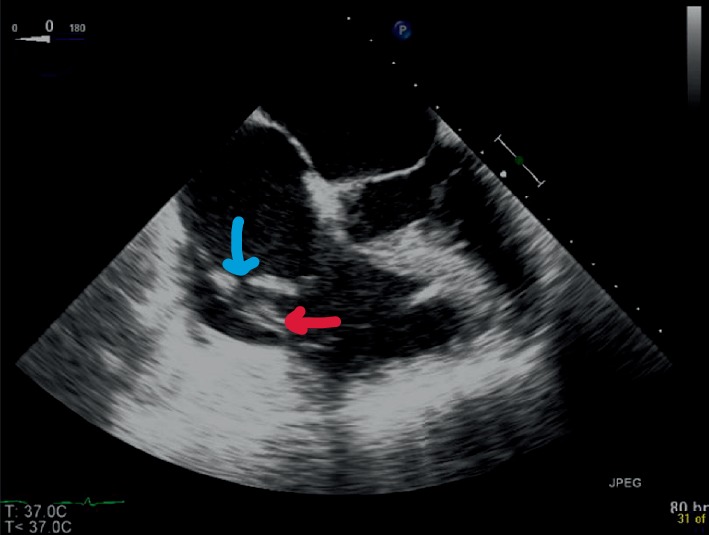
Transesophageal echocardiogram at the midesophageal four chamber view showing rupture of the papillary muscle (red arrow) resulting in protrusion of the tricuspid valve and subvalvular apparatus into the right ventricle, and detached anterior leaflet from the annulus (blue arrow). Significant dilatation of right atrium, with increased pressure as demonstrated by the bowing of the interatrial septum into the left atrium.

The patient was taken emergently to the operating room for exploration and veno-arterial extracorporeal membrane oxygenation (VA ECMO) cannulation. With worsening hemodynamics and increasing inotropic and pressor support (including intravenous epinephrine 0.07–0.1 mcg/kg/min, addition of milrinone 0.5 mcg/kg/min, vasopressin 0.04 units/min–0.08 units/min, and norepinephrine none to 8 mg/min), an emergent TEE was performed. The intraoperative TEE demonstrated poor biventricular function with severe tricuspid regurgitation and papillary rupture (Figure 1).

With the extensive chest wall injuries, VA ECMO was thought to be contraindicated given the need for systemic anticoagulation and concern for hemorrhage. Chest wall bleeding was stabilized and the patient was taken to the ICU with his chest packed open. The postoperative course was complicated by profound hypotension, hepatorenal failure, and persistent pulsatile ventricular tachycardia requiring multiple cardioversions and amiodarone infusion. Once hemodynamically stabilized on hospital day 2, the patient returned to the operating room for continued control of chest wall hemorrhage.

**Figure 2:**
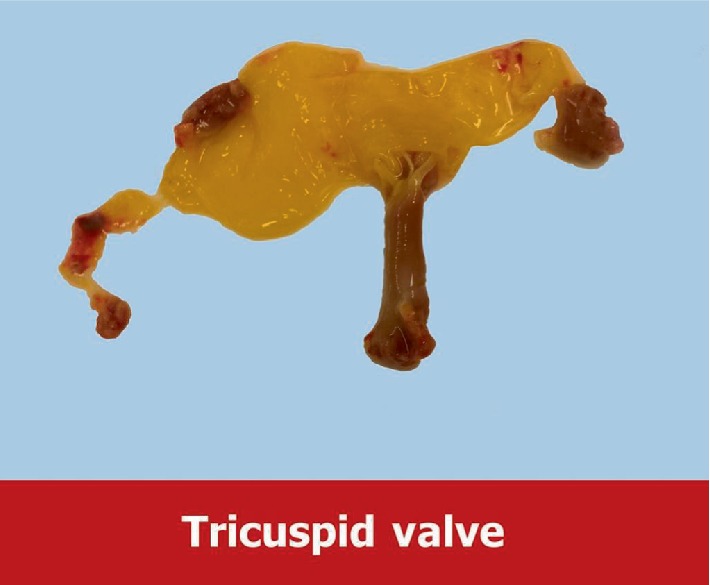
Photograph of the pathology specimen of the excised ruptured tricuspid valve.

On hospital day 3, the patient underwent clamshell thoracotomy and tricuspid valve replacement using a beating heart technique. The patient remained intubated from the initial presentation. His access lines included a left subclavian multi lumen access (MAC) catheter, and right radial arterial line. Anesthesia was maintained with a combination of volatile and intravenous agents combined with boluses of intravenous narcotics. His prebypass TEE showed an ejection fraction of 55% with an underfilled left ventricle and septal flattening. There was severe right atrial and right ventricular dilatation. The tricuspid valve had flail anterior and posterior leaflets. Full heparinization and central aortic and bicaval cannulation were achieved. On cardiopulmonary bypass, the right atrium was opened and the valvular anatomy was inspected (Figure 2). The anterior tricuspid leaflet was completely detached. The annulus was severely dilated (54 mm). The valvular anatomy was unable to be repaired. A tricuspid valve replacement was performed using a #29 Edwards pericardial tissue valve with a cardiopulmonary bypass time of 70 minutes. The competence of the new valve was tested by injecting saline into the right ventricle and confirmed by TEE after coming off bypass. The right atrium was closed and the patient weaned from cardiopulmonary bypass. Post bypass TEE demonstrated no residual tricuspid regurgitation, good leaflet coaptation and no perivalvular leak. Biventricular ejection fraction remained low. The patient required continued significant inotropic and pressor support.

Despite surgical intervention, the patient remained in cardiogenic shock requiring escalating doses of epinephrine, vasopressin, milrinone and inhaled epoprostenol sodium (Flolan). He succumbed to his injuries on hospital day 5 and expired.

## 3. Discussion

Cardiac trauma from non-penetrating chest injuries is uncommon, with an estimated incidence of 30,000 cases per year in the United States [7]. These injuries typically involve contusion of the myocardium, but can involve valvular structures, including the tricuspid valve [8].

As the right ventricle is located immediately posterior to the sternum, the tricuspid valve is prone to compressive injuries. Increases in thoracic pressure can cause increases in right ventricular pressure during end diastole, when the tricuspid valve is closed, resulting in increased forces on the valvular and subvalvular apparatus. Sudden and violent deceleration forces can cause rupture of the leaflet, chordae tendinae or papillary muscles leading to severe tricuspid insufficiency [9]. According to this report, the most frequent site of injury is the tendinous cords, followed by the anterior papillary muscle and tear or detachment of the anterior leaflet.

Tricuspid valve injury may go undetected during the initial evaluation of trauma patients. Often during the initial survey, life threatening injuries take precedence, and many cases of traumatic tricuspid insufficiency are not detected during early presentation. In addition, tricuspid regurgitation may be well tolerated with minimal clinical manifestations until progressive deterioration of right heart function occurs, causing increased shortness of breath, arrhythmias and chest pain [8, 10]. Therefore delays in diagnosis can lead to a delay in treatment.

Clearly this patient presented with evidence of significant blunt chest trauma. However, the initial FAST examination did not diagnose a ruptured tricuspid valve. Although sensitive and usually accurate, occasional false negative results do occur especially when cardiac injury does not result in accumulation of intra-pericardial fluid [11]. It is important to stress the importance of exploring other diagnostic modalities when faced with significant blunt chest trauma and considering the diagnosis of cardiac injury despite negative FAST examination [12]. At this stage with a high index of suspicion, a transthoracic echocardiogram, transesophageal echocardiogram, magnetic resonance imaging (MRI) or a more refined interrogation with FAST should have confirmed this diagnosis and can provide valuable information regarding right ventricular function, and functional characteristics of the valve [13]. All of this information is important in determining the appropriate planning for the surgical procedure.

Interestingly, in this case, there was a relatively slow deterioration with development of right sided heart failure which is usually seen in more chronic conditions and can be tolerated for a relatively long time. Sudden rupture of the tricuspid valve is not usually tolerated, and patients deteriorate with a matter of hours. Severe valvular dysfunction usually results in rapid clinical deterioration resembling cardiac tamponade with hemodynamic instability that is difficult to treat. An echocardiogram would be diagnostic of this condition.

When the clinicians failed to discover tamponade in the setting of right sided distention, it should have prompted the surgeons to explore other potential diagnoses. In this case, the clinicians were focused on the patient's other injuries and had ruled out suspected aortic injury. Suspicion for tricuspid valve injury was missed despite the mechanism of injury, murmur and right sided cardiac contusion. Calling for an intraoperative transesophageal echocardiogram or even an intraoperative consult with a cardiac surgeon would have confirmed the diagnosis and allowed for a timely tricuspid valve replacement.

The question was also raised regarding systemic anticoagulation needed for institution of VA ECMO or even surgical replacement of the tricuspid valve. While surgical replacement would have been the definitive management for this condition, persistent chest wall hemorrhage, rapidly progressive hepatorenal syndrome (with low platelet count and abnormal coagulation panel), and worsening patient instability raised the concern regarding whether the patient would tolerate these maneuvers. The attending trauma surgeon elected to delay definitive repair until the patient was more hemodynamically stable hoping that his chance for survival would improve.

## 4. Conclusion

We describe a unique case of a post traumatic avulsion of the tricuspid valve which is a rare complication of chest trauma. A high index of suspicion should have resulted in an earlier diagnosis. Development of sudden and profound right heart failure necessitates diagnosis and repair as quickly as feasible, before irreversible end-organ damage occurs. Extra-corporeal membrane oxygenation can be considered as a temporizing measure for hemodynamic and ventilatory support in clinically relevant cases. Tricuspid valve repair or replacement is the only surgical option and the decision will be made based on the extent of valve damage. Without surgical intervention, severe tricuspid regurgitation may result in right ventricular failure, progressive congestive heart failure, and untimely demise.

## References

[1] Khanna S. N., Paul M., Sharma R., Sharma K. K. (2014). Repair of tricuspid insufficiency following blunt trauma chest-a case report and review of literature. *Journal of Cardiovascular Diseases & Diagnosis*.

[2] Mehrotra D., Dalley P., Mahon B. (2012). Tricuspid valve avulsion after blunt chest trauma. *Texas Heart Institute Journal*.

[3] Song H. J., Nam S. H., Choi Y. J., Park S. H., Park S. H., Han J. J. (2004). A case of native valve salvage for 8 years longstanding ruptured tricuspid valve after blunt chest trauma. *Korean Circulation Journal*.

[4] Schuster I., Graf S., Klaar U., Seitelberger R., Mundigler G., Binder T. (2008). Heterogeneity of traumatic injury of the tricuspid valve: a report of four cases. *Wiener klinische Wochenschrift *.

[5] Nelson M., Wells G. (2007). A case of traumatic tricuspid valve regurgitation caused by blunt chest trauma. *Journal of the American Society of Echocardiography*.

[6] Shaikh N., Ummunissa F., Sattar M. A. (2013). Traumatic mitral valve pericardial injury. *Case Reports in Critical Care*.

[7] Elie M. C. (2006). Blunt cardiac injury. *The Mount Sinai Journal of Medicine*.

[8] Chirillo F., Totis O., Cavarzerani A. (1996). Usefulness of transthoracic and transoesophageal echocardiography in recognition and management of cardiovascular inju- ries after blunt chest trauma. *Heart*.

[9] Avegliano G., Corneli M., Conde D., Ronderos R. (2014). Traumatic rupture of the tricuspid valve and multi-modality imaging. *Cardiovascular Diagnosis and Therapy*.

[10] Theodoropoulos I., Cheeyandira A., Tortella B. (2013). Traumatic tricuspid valve rupture presenting as third-degree atrioventricular block. *The Journal of Emergency Medicine*.

[11] Baker L., Amadani A., Ball C. G. (2015). False negative pericardial focused assessment with sonography for trauma examination following cardiac rupture from blunt thoracic trauma: a case report. *Journal of Medical Case Reports*.

[12] Nan Y.-Y., Lu M.-S., Liu K.-S. (2009). Blunt traumatic cardiac rupture: therapeutic options and outcomes. *Injury*.

[13] Khurana S., Puri R., Wong D. (2009). Latent tricuspid valve rupture after motor vehicle accident and routine echocardiography in all chest-wall traumas. *Texas Heart Institute Journal*.

